# Thyroglobulin doubling time offers a better threshold than thyroglobulin level for selecting optimal candidates to undergo localizing [^18^F]FDG PET/CT in non-iodine avid differentiated thyroid carcinoma

**DOI:** 10.1007/s00259-020-04992-8

**Published:** 2020-08-13

**Authors:** Domenico Albano, Mark Tulchinsky, Francesco Dondi, Angelica Mazzoletti, Davide Lombardi, Francesco Bertagna, Raffaele Giubbini

**Affiliations:** 1 Nuclear Medicine, University of Brescia and Spedali Civili Brescia, P.le Spedali Civili 1, 25123 Brescia, Italy; 2 Section of Nuclear Medicine, Department of Radiology, Milton S. Hershey Medical Center, Penn State Health, Hershey, PA USA; 3 Department of Otorhinolaryngology - Head and Neck Surgery, University of Brescia, Brescia, Italy

**Keywords:** Thyroglobulin doubling time, Differentiated thyroid carcinoma, Thyroglobulin, [^18^F]FDG PET/CT, Diagnostic test performance

## Abstract

**Purpose:**

To investigate the potential role of serum thyroglobulin doubling time (TgDT) in predicting 2-deoxy-2-[^18^F]fluoro-d-glucose ([^18^F]FDG) PET/CT results in patients affected by differentiated thyroid carcinoma (DTC) who demonstrated a combination of positive Tg but a negative [^131^I] whole-body scan ([^131^I]-WBS).

**Materials and methods:**

Inclusion criteria were (1) prior [^131^I] treatment for DTC, (2) negative subsequent [^131^I]-WBS, (3) no interfering anti-Tg antibodies, (4) three consecutive Tg measurements under the thyroid hormone replacement therapy to calculate TgDT before 2-[^18^F]FDG PET/CT, and (5) at least 6 months of clinical and/or imaging follow-up to ascertain the diagnosis. Receiver operating characteristic (ROC) curve and the area under the curve (AUC) were used to identify the optimal cutoff point for the last stimulated Tg and TgDT prior to [^18^F]FDG PET/CT.

**Results:**

One hundred and thirteen patients were included. Seventy-four (65%) patients had positive [^18^F]FDG PET/CT for DTC recurrence, while the remaining 39 (35%) negative. Sensitivity, specificity, positive predictive value, negative predictive value, and accuracy of [^18^F]FDG PET/CT were 92%, 94%, 97%, 87%, and 93%. Patients with positive [^18^F]FDG PET/CT had higher Tg and TgDT than those with negative PET/CT. ROC curve analysis revealed an optimal Tg cutoff of 19 ng/mL (sensitivity 78%, specificity 85%, AUC = 0.844) and TgDT of 2.5 years (sensitivity 93%, specificity 87%, AUC = 0.911). TgDT threshold of 2.5 years predicted significantly (*p* = 0.023) better than Tg level PET/CT results.

**Conclusions:**

The diagnostic performance of [^18^F]FDG PET/CT could be significantly improved when TgDT is less than or equal to 2.5 years, as compared with using the absolute Tg level.

## Introduction

Differentiated thyroid cancer (DTC) is the most frequent endocrine cancer, which is usually characterized by a favorable outcome and a long-term survival [1] in patients without distant metastases [2]. The initial therapy consists of total thyroidectomy followed by postoperative sodium iodide [^131^I] ([^131^I]) treatment in most cases. Serum thyroglobulin (Tg) is a sensitive tumor marker used in detection of residual and/or surveillance for recurrent DTC. In case of suspected recurrence, a neck ultrasound and a diagnostic [^131^I] whole-body scan ([^131^I]-WBS) are the initial examinations for localizing and staging the disease [3], but they have some limitations. Ultrasound is an operator-dependent technique and limited to the study of cervical lymph node metastases only, while [^131^I]-WBS is able to detect only the iodine avid DTC. The 2-deoxy-2-[^18^F]fluoro-d-glucose positron emission tomography/computed tomography ([^18^F]FDG PET/CT) is able to identify metastatic DTC throughout the body and does not rely on iodine avidity. In fact, the main indication for [^18^F]FDG PET/CT in DTC is to localize non-iodine avid recurrent or residual disease in patients with detectable (usually > 1.0 ng/mL) or increasing Tg levels who had a negative [^131^I]-WBS and evaluation of prognosis and assessment/definition of RAI refractory disease [4–6].

Moreover, [^18^F]FDG PET/CT is a safe and sophisticated diagnostic modality but it is also costly and associated with radiation exposure. Therefore, it requires a judicious utilization that should be optimized for diagnostic yield. There is a wide range of proposed Tg thresholds to achieve optimal [^18^F]FDG PET/CT accuracy in patients with DTC [7–9]. One study [10] suggested that Tg doubling time (TgDT) may be independently useful. We aimed to investigate the threshold levels for Tg and TgDT that best correlate with the [^18^F]FDG PET/CT diagnostic performance and compare their relative usefulness for localizing sites of non-iodine avid DTC in patients with detectable Tg but a negative [^131^I]-WBS.

## Materials and methods

### Patients

**Fig. 1:**
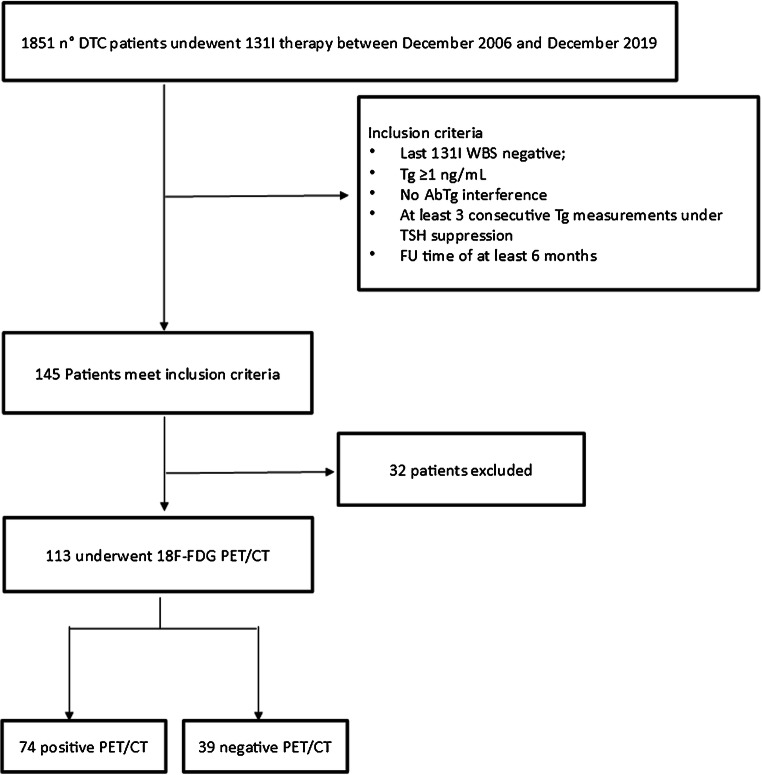
Flow-chart of the cohort selection

From December 2006 to December 2019, among 1851 patients affected by DTC who were treated with total thyroidectomy plus ^131^I DTC, 113 patients (median age 56 years, 61 female, 52 male) with last post-therapeutic [^131^I]-WBS negative but Tg ≥ 1 ng/mL without detectable Tg antibodies underwent [^18^F]FDG PET/CT and were retrospectively included in this study (Fig. 1). Pertinent clinico-pathologic characteristics are included in Table 1. They were admitted to our Nuclear Medicine Department for the ablation of thyroid remnant and/or therapy according to the EANM (European Association of Nuclear Medicine) guidelines [11]. The administered activity of ^131^I first ablation treatments ranged from 1.1 to 5.5 GBq (average 3.2 GBq) and it was established according to the risk class based on the TNM staging of the American Joint Committee on Cancer/International Union Against Cancer currently in use and the status of the disease. Ninety-six patients underwent levothyroxine withdrawal for 40 days, replaced by triiodothyronine in the first 20 days, and in seventeen patients, recombinant human thyrotropin (rhTSH) (Genzyme Corporation) was administered intramuscularly with a dose of 0.9 mg on 2 consecutive days during treatment with levothyroxine, and radioiodine was administered the day after the second injection. Every patient followed a low-iodine diet for at least 2 weeks and the serum thyrotropin (TSH) level was higher than 30 m UI/L before ^131^I administration.

**Table 1:** Baseline features of our population

	Average (range)	Patients, *n* (%)
Age years	54 (10–82)	
Male		52 (46%)
Female		61 (64%)
Histotype		
Papillary		55 (49%)
Follicular variant of papillary		16 (14%)
Follicular		19 (17%)
Aggressive variant		15 (13%)
Hurtle cell		7 (6%)
Unknown (Tx)		1 (1%)
Tumor size (mm)	32 (5–90)	
Multicentricity		37 (32%)
Thyroiditis		13 (11%)
T-stage		
sTx		1 (1%)
sT1		18 (16%)
sT2		28 (25%)
sT3		51 (45%)
sT4		15 (13%)
N-stage		
sN0		64 (57%)
sN1a		24 (21%)
sN1b		25 (22%)
M-stage		
sM1		8 (7%)
ATA class risk low:intermediate:high		21:72:20
Tg at the time of ablation (ng/mL)	186 (0.1–1001)	
TgAb positive at ablation		24 (21%)
First RAI activities administrated (GBq)	3.2 (1–5.5)	
Cumulative RAI activities administrated (GBq)	29.8 (1.1–74.6)	
N° therapies	4.4 (1–10)	

*n* number, *GBq* gigabecquerel, *RAI* radioiodine, *var* variant, *Tg* thyroglobulin, *Ab* antibodies

### [^131^]I Whole-body imaging

All patients underwent [^131^I]-WBS followed by single-photon emission computed tomography/computed tomography (SPECT/CT) scanning 3–4 days after [^131^I] administration. Hybrid SPECT/CT scans were routinely performed from skull base to the lung bases and additional SPECT/CT scans of other areas were performed, depending on [^131^I]-WBS findings. The WBS was performed in continuous mode with high-energy general purpose (HEGP) parallel hole collimator, 364-keV photopeak with ± 10% energy windows setting and scatter correction. The infrared-based real-time automatic body contouring system was activated for simultaneous dual-view (anterior/posterior) scans with a matrix of 256 × 1024. The scan speed was 11 cm/min for injected activities ranging from 1100 to 1850 MBq and 15 cm/min for 3700 Mbq. On the same day, a hybrid dual-detector SPECT/CT (Infinia Hawkeye II, GE Healthcare, Haifa Israel) equipped with 1-in. StarBrite™ Crystal and a high-energy collimator was performed. The StarBrite™ Crystal has a sensitivity more than twofold higher than that provided by a 3/8-in. crystal without a significant loss in resolution, therefore contributing to improve the reliability of SPECT with ^131^I. SPECT images were acquired with HEGP collimator, matrix size of 128 × 128, 364-keV photopeak with ± 10% energy and scatter windows, dual-detector 180° acquisition, angular step of 3°, and 15′′ time per step/view. The CT parameters were 140 kV, 2.5 mA, 30′′ rotation speed, 10-mm slice thickness, and 256 × 256 matrix. CT acquisition was performed with a 2-slice helicoidal acquisition. An ordered subset expectation maximization iterative reconstruction with CT-based attenuation correction and scatter correction was performed.

### Thyroglobulin-related evaluation

Tg was measured using an immunoradiometric assay (DYNOtest® Tg-plus; BRAHMS Diagnostica, Hennigsdorf, Germany) according to the manufacturer’s instructions. The presence of autoantibodies against Tg was evaluated by a specific radioimmunoassay (DYNOtest® anti-Tg_n_; BRAHMS Diagnostica, Hennigsdorf, Germany).

The TgDT was computed on the basis of at least three consecutive Tg measurements under TSH suppression treatment with all values obtained prior to the PET/CT scan. This calculation followed the procedure by Kuma Hospital (https://www.kuma-h.or.jp/english/about/doubling-time-progression-calculator/). Aside from TgDT, we analyzed also the last stimulated Tg before [^18^F]FDG PET/CT scan, measured in all cases after thyroid hormone withdrawal.

### [^18^F]FDG PET/CT imaging and interpretation

All [^18^F]FDG PET/CT scans were performed following the international guidelines of the European Association of Nuclear Medicine [12]. The patients underwent [^18^F]FDG PET/CT after at least 6 h of fasting and with glucose level lower than 160 mg/dL. An activity of 3.5–4.5 MBq/kg of [^18^F]FDG was injected intravenously and scans were acquired about 60 min after radiotracer injection from the skull base to the mid-thigh on a Discovery ST or 690 PET/CT tomograph (General Electric Company—GER—Milwaukee, USA) with standard parameters (CT: 80 mA, 120 kV without contrast; 2.5–3.5 min per bed-PET-step of 15 cm); the reconstruction was performed in a 128 × 128 or 256 × 256 matrix and 60 cm field of view. In our center, PET/CT was performed without contrast due to local and organizational reasons.

The PET/CT images were reviewed by an expert nuclear medicine physician (FB) and every focal tracer uptake different from physiological background and with activity higher than the surrounding tissue was considered as suggestive of disease. For the evaluation of accuracy of PET/CT results, a combination of clinical and/or imaging follow-up for at least 6 months was taken as reference standard, including cytological reports, histopathologic examinations, and further imaging studies such as CT with or without contrast, ultrasound, and magnetic resonance imaging. In addition, the rising Tg trend over 6 months was accepted as a biochemical indication of disease. Due to the fact that histopathological confirmation of all lesions was not ethically and clinically feasible in every case, histopathology was obtained in 40 patients where it was warranted. Positive [^18^F]FDG PET/CT findings were judged true-positive when DTC was confirmed by cytology or histopathology or when a subsequent imaging technique detected the same lesions together with increased Tg level. Positive [^18^F]FDG PET/CT findings were defined as false-positive when cytology or histopathology excluded DTC or when the lesions had resolved on subsequent follow-up imaging without treatment. Negative [^18^F]FDG PET/CT was conventionally deemed as true-negative when recurrences/structural diseases were not demonstrable during the whole subsequent follow-up with a stable or decreasing Tg level. Negative [^18^F]FDG PET/CT was judged as false-negative if cytology or histological examination showed metastatic DTC or when progression of disease was demonstrated on other imaging modalities in combination with increasing Tg. The mean follow-up time was 51 months (range 6–120 months); for patients with negative PET/CT scan, average follow-up time was 60 (range 24–99).

### Statistical analysis

Statistical analyses were performed using MedCalc Software version 18.1. The descriptive analysis of categorical variables is characterized by the calculation of simple and relative frequencies, while the numeric variables by median, mean, minimum, and maximum values. Using final clinico-pathologic diagnosis as a reference, sensitivity, specificity, positive predictive value (PPV), negative predictive value (NPV), and accuracy were calculated based on Bayes’ law and supplemented with 95% confidence intervals (CIs). The Tg and TgDT were assessed as having normal distribution by the Shapiro-Wilk test. The Mann–Whitney *U* test was used to compare the distribution of variance in different groups. Differences between categorical values were assessed using the two-tailed chi-squared test. A *P* value < 0.05 was considered to indicate statistical significance.

For the entire population, receiver operating characteristic (ROC) curve analysis was used to identify the optimal cutoff point of Tg and TgDT in the light of which interpret the results of [^18^F]FDG PET/CT. ROC curves were compared using the nonparametric method described by DeLong.

## Results

**Fig. 2:**
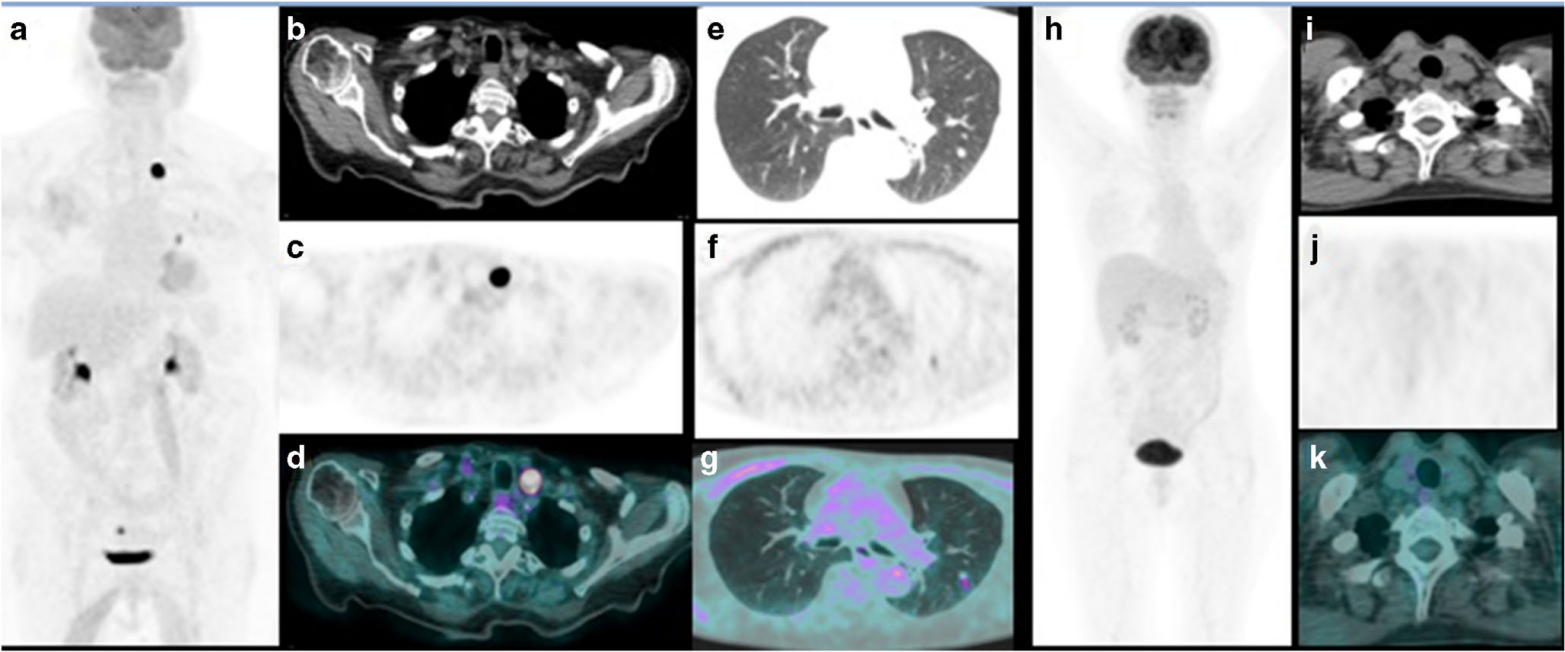
A representative case of a 71-year-old female with a conventional papillary carcinoma (pT3N1b according to AJCC 8th edition) who was imaged with [^18^F]FDG PET/CT while showing Tg of 6 ng/mL and TgDT of 1.12 years. Maximum intensity projection (MIP, **a**) showing several focal hypermetabolic lesions in the neck and chest. Axial CT (**b**), PET (**c**), and PET/CT (**d**) fused scan showing an increased FDG uptake in the left thyroid bed consistent of relapse. Thoracic CT (**e**), PET (**f**), and PET/CT (**g**) demonstrating a small lung nodule in the left superior lobe FDG-avid. Another case of a 49-year-old woman with follicular carcinoma (pT1bN0) with a negative [^18^F]FDG PET/CT (**h**). Neck CT (**i**), PET (**j**), and PET/CT (**k**) fused images showing no hypermetabolic lesions and Tg of 6 ng/mL but TgDT of 7.64 years

Among 113 [^18^F]FDG PET/CT scans, 74 (65%) were interpreted as positive for disease based on showing the presence of hypermetabolic lesions **(**Fig. 2). Of these, 72 were true-positive. The presence of disease was established by fine-needle aspiration in 6 cases (2 in thyroid bed and 4 in cervical nodes) and by post-surgical histopathology in 20 (2 in thyroid bed, 4 in thyroid bed plus cervical nodes, 12 only in cervical nodes, and 2 in lung metastases). The remaining 46 true-positive examinations were confirmed by compelling clinical and/or radiological and/or biochemical evidence of progression (20 in lungs, 10 in bones, 7 in coexisting lymph node and lung and/or bone metastases, 5 in coexisting bone and lung metastases, 1 in coexisting lung plus bone plus liver metastases, 1 in muscle, 1 in liver, and 1 in brain metastases). In the two false-positive [^18^F]FDG PET/CT examinations, one patient had a cervical FDG-avid lymph node that revealed a benign cytology on biopsy and the other patient had an FDG-avid lung nodule that resolved spontaneously on follow-up.

There were 39 (35%) patients with negative [^18^F]FDG PET/CT examinations. Of these, 33 were true-negative because no structural disease was detected in the subsequent follow-up imaging examinations. There were 6 false-negative [^18^F]FDG PET/CT examinations that included four patients with sub-centimeter lung nodules detected on a high-resolution CT that were below PET/CT resolution by size criteria and two patients who demonstrated biochemical disease progression.

The overall [^18^F]FDG PET/CT sensitivity, specificity, PPV, NPV, accuracy, and positive and negative likelihood ratios were 92% (95% CI 84–97%), 94% (95% CI 81–99%), 97% (95% CI 90–99%), 87% (95% CI 72–92%), 93% (95% CI 87–97%), and 16.15 and 0.08, respectively. The stimulated Tg levels at the time of PET/CT were significantly higher in patients with a positive study (median 69.5 ng/mL, mean 156 ng/mL, range 1.2–1001 ng/mL) than in patients with a negative PET/CT (median 12 ng/mL, mean 18 ng/mL, range 1.1–89 ng/mL; *p* < 0.001). TgDT was significantly higher in patients with negative PET/CT than that with positive (median 4.52 year, mean 8.9 years, range 0.3–110 vs median 1.07 year, mean 1.3, range 0.12–24; *p* < 0.001) (Table 2).

**Table 2:** Comparison between patients with positive and negative [^18^F]FDG PET/CT

	Positive [^18^F]FDG PET/CT, *n* 74	Negative [^18^F]FDG PET/CT, *n* 39	*P* value
Gender F:M	39:35	22:17	0.709
Age, mean ± DS	55.7 ± 16	50.4 ± 14	0.166
Histotype			0.004
Papillary	30 (41%)	25 (64%)	
Follicular var. of papillary	9 (12%)	7 (18%)	
Follicular	15 (20%)	4 (10%)	
Aggressive variant	12 (16%)	3 (8%)	
Hurthle cell	7 (9%)	0 (0%)	
Tumor size (mm), mean ± DS	34.3 ± 16.6	28.4 ± 20.7	0.108
TNM			0.225
Tx, 1, 2	28 (38%)	19 (49%)	
T3, T4	46 (62%)	20 (51%)	
Multicentricity	21 (28%)	16 (41%)	0.245
First ^131^I activity (GBq), mean ± DS	3.2 ± 1	3.1 ± 1.3	0.944
Cumulative ^131^I activities (GBq), mean ± DS	30.5 ± 16.8	28.4 ± 16.7	0.533
Tg at the time of PET/CT (ng/mL), mean ± DS	156 ± 133	18 ± 15	< 0.001
Tg ≤ 19 ng/mL	16 (22%)	30 (77%)	< 0.001
Tg >19 ng/mL	58 (78%)	9 (23%)	
TgDT years, mean ± DS	1.3 ± 0.9	8.9 ± 19.2	< 0.001
TgDT ≤ 2.5 years	68 (92%)	5 (13%)	< 0.001
TgDT >2.5 years	6 (8%)	34 (87%)	

*F* female, *M* male, *Tg* thyroglobulin, *TgDT* Tg doubling time, *DS* deviation standard

**Fig. 3:**
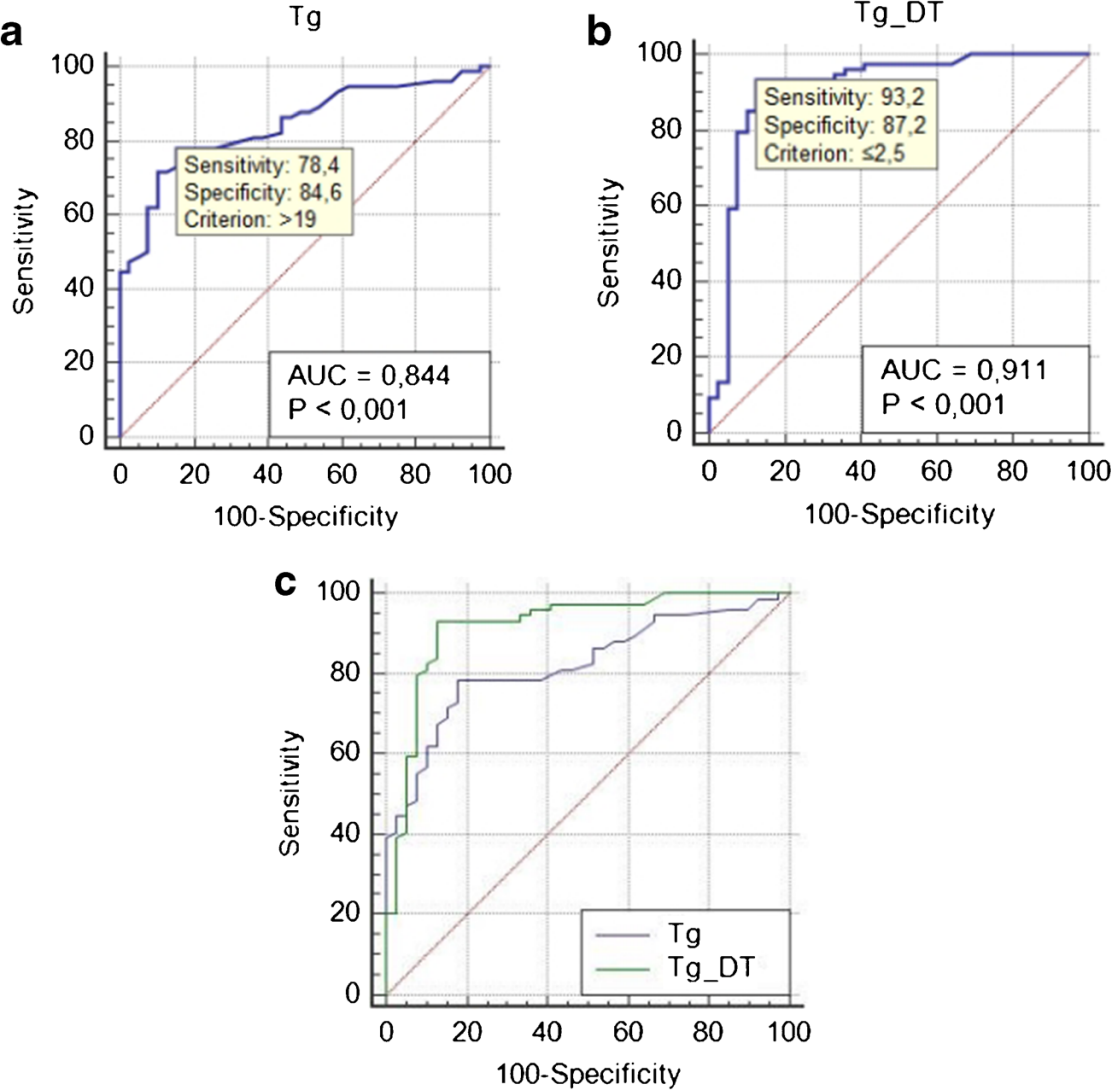
ROC curve analyses which evaluated the relationship between Tg in ng/mL (**a**) and TgDT in years (**b**) thresholds for identifying positive PET/CT results. Comparison of ROC curves shows better characteristics for thresholding with TgDT compared with Tg (**c**)

The ROC curve analysis (Fig. 3a, b) revealed an optimal stimulated Tg cutoff of 19 ng/mL and TgDT cutoff of 2.5 years for predicting the best PET/CT diagnostic performance. The Tg cutoff of 19 ng/mL correctly predicted 57 of 72 (79%) true-positive and 30 of 33 (91%) true-negative PET/CTs, while the TgDT threshold of 2.5 years predicted 66 of 72 (92%) true-positive and 31 of 33 (94%) true-negative PET/CTs. In fact, among 72 patients with true-positive PET/CT, 66 (92%) had TgDT ≤ 2.5 years (average of 1.08 years and range of 0.12–2.36 year) and among 33 patients with true-negative PET/CT, 31 (94%) had TgDT > 2.5 years. The ROC for TgDT was 0.0981 greater than for Tg, which was statistically significant (*p* = 0.023) (Fig. 3). In the patient with false-positive PET/CT scan, TgDT was higher than 2.5 years, while Tg was 50 ng/mL. Of six false-negative PET/CT scans, only two had TgDT > 2.5 years, while three had Tg < 19 ng/mL.

Considering the clinico-pathologic features, only histopathology was significantly different between positive and negative [^18^F]FDG PET/CTs with more of aggressive and Hurthle cell variants in patients with positive PET/CT (Table 2).

## Discussion

Tg is produced specifically by normal thyrocytes and/or by a majority of DTC cells, which underpins its utilization as specific marker of residual, persistent, and recurrent DTC [13–15]. It is a very sensitive marker and its periodical measurement after initial adjuvant/ablative thyroid [^131^I] is recommended. However, Tg measurements could be influenced by several variables, including the sensitivity of the assay method [16], TSH level [17], degree of DTC differentiation, and presence of heterophile antibodies and anti-Tg antibodies [18]. It is not so rare that in some patients with low but detectable Tg levels (i.e., biochemical evidence of disease) the diagnostic imaging is negative (i.e., no evidence of structural disease). In fewer cases, the imaging demonstrates residual/recurrent DTC associated with a false-negative Tg testing because the tumor dedifferentiated and lost its ability to produce Tg.

Unlike static Tg testing, there is paucity of clinical guidelines about Tg kinetics, which can be measured by TgDT and/or Tg velocity (TgV). Miyauchi et al. [19] first suggested that TgDT could predict locoregional recurrence, distant metastases, and disease specific survival. They tested four categories of TgDT dynamics using arbitrary cutoffs: < 1 year for rapidly growing, 1 to 3 years for slowly growing, > 3 years for stable, and negative TgDT for improving disease. These categories performed better for prognostication of DTC patients than classic variables, such as age and primary’s size. These authors stressed the point about exponential changes in volume of growing tumors, which is most amenable to measurements with doubling time rather than the alternative of velocity (best for linear changes). Subsequent papers [20, 21] confirmed these findings. Interestingly, TgV also showed prognostic value for predicting DTC recurrence, demonstrating a specificity of 94.4% and a sensitivity of 83.3% at the threshold of 0.3 ng/mL/year. In addition, patients with TgV below 0.3 ng/mL/year showed a significantly better overall survival [22].

It is logical to anticipate that rapidly growing tumors should have a greater metabolic activity and are more likely to exhibit FDG avidity. Indeed, a study by Kelders et al. [23] found an association between a TgDT of < 1 year and a positive [^18^F]FDG PET/CT for metastases, while the [^131^I]-WBS was negative. They also found that eight of nine patients with short TgDT had FDG-avid but [^131^I]-negative DTC exhibited a poor prognosis [23]. But despite these results, TgDT did not seem to predict survival in patients with radioiodine refractory DTC who were treated with tyrosine kinase inhibitors [24, 25]. Manohar et al. [26] showed in their cohort of patients with distant DTC metastases that a suppressed absolute thyroglobulin Tg of > 100 ng/mL was a stronger prognostic marker for progression-free survival and overall survival than the TgDT at a threshold of < 6 months. The same study used metabolic indexes from [^18^F]FDG PET/CT that were even more powerful predictors of the same outcomes, concluding that the imaging contributed to dynamic prognostication in this population. Indirectly, the study makes another logical link between a rapidly growing DTC by TgDT metric and metabolic activity on [^18^F]FDG PET/CT.

In spite of this strong logical link between the TgDT and [^18^F]FDG PET/CT, the use of TgDT in selecting optimal candidates for [^18^F]FDG PET/CT imaging to improve its diagnostic yield has been investigated in only one paper [10]. That study demonstrated that the accuracy of [^18^F]FDG PET/CT was significantly and independently improved when the test was reserved for those with unstimulated serum Tg above 5.5 ng/mL and the TgDT of less than 1 year. The study included 102 patients with DTC who had detectable Tg after [^131^I] treatment. In our analysis of 113 patients with DTC, we found that the best cutoff for TgDT was 2.5 years, predicting a positive [^18^F]FDG PET/CT with sensitivity and specificity of 93.2% and 87.2%, respectively (AUC of 0.911). Furthermore, the best threshold for absolute Tg was at 19 ng/mL, but AUC analysis showed it significantly (*p* = 0.023) less predictive for identifying patients with a positive [^18^F]FDG PET/CT examination (AUC = 0.844), as shown in Fig. 3c. Therefore, TgDT at the threshold of 2.5 and less assures the best diagnostic yield [^18^F]FDG PET/CT in DTC patients with detectable Tg but a negative [^131^I]-WBS.

In the prior studies, TgDT cutoff was arbitrarily selected [10, 17]. We improved the methodology of determining the threshold by using ROC curve analysis, which is usually used for this kind of analysis. It is also applicable to statistical comparison of various tests that use threshold approach for dichotomizing the cohort. This allowed us to conclude with good level of certainty that TgDT is a better marker for selecting DTC patients for additional imaging with [^18^F]FDG PET/CT when the [^131^I]-WBS is negative. Further studies are needed to validate our results in a larger cohort at a different institution to validate the identified TgDT threshold of 2.5 years.

Our study contains some limitations, including its retrospective design, the absence of prognostic analysis, the potential use of heterogeneous management approaches over a relatively long period included, and the absence of histological confirmation of all lesions. Another limitation is the difficult practicability of TgDT in clinical practice related to the need of three consecutive Tg measurements in similar condition (under TSH suppression treatment), possibly not distant in time.

This issue may be partially attenuated by those centers that followed patients regularly with controls every 6 months.

However, the outcome of patients was not the goal of our investigation as we aimed to optimize selection of a specific segment of DTC patients for further imaging with [^18^F]FDG PET/CT in order to localize the source of elevated Tg in the face of a negative [^131^I]-WBS, i.e., non-iodine avid DTC. The real question now is whether this approach could expedite removal of occult metastatic disease and thereby improve the outcomes of such patients. This exciting question deserves our further attention and prospective investigation.
